# Supplementary material for: DDK regulates replication initiation by controlling the multiplicity of Cdc45-GINS binding to Mcm2-7
Source: eLife. 2021 Feb 22;10:e65471. doi: 10.7554/eLife.65471 (PMC7954526; doi:10.7554/eLife.65471)
Supplement: Supplementary file 1. — Supplementary file 1a. Fit parameters for Cdc45 fluorescence-intensity histograms. Supplementary file 1b. Fit parameters for GINS fluorescence-intensity histograms. [file elife-65471-supp1.docx]

Supplementary Files

for

DDK regulates replication initiation by controlling

the multiplicity of Cdc45-GINS binding to Mcm2-7

Lorraine De Jesús-Kim^1^, Larry J. Friedman^2^, Marko Lõoke^1,3^, Christian K. Ramsoomair^1^,

Jeff Gelles^2, *^, and Stephen P. Bell^1, *^

^1^Howard Hughes Medical Institute, Department of Biology, Massachusetts Institute of Technology, Cambridge, MA 02139, USA

^2^Department of Biochemistry, Brandeis University, Waltham, MA 02454, USA

^3^Current address: Institute of Technology, University of Tartu, Tartu 50411, Estonia

^*^Co-corresponding authors:

Stephen P. Bell

email: spbell@mit.edu

phone: 617-253-2054

Jeff Gelles

email: gelles@brandeis.edu

phone: 781-736-2377

**Supplementary File 1a. Parameters used for fitting of Cdc45 fluorescence-intensity histograms.**

| **Fit parameters^b^**  **[90% CI]** | | | | **Fraction of time that DNAs with loaded Mcm2-7^4SNAP549^ were bound by this number of Cdc45^SORT649^ molecules (± SE)^d^** | | | | | |  |  |
| --- | --- | --- | --- | --- | --- | --- | --- | --- | --- | --- | --- |
| **Condition**  **(Figure)** | ***q*** | ***s***  **(× 10^-3^)** | **λ** | ***x***  **(× 10^-3^)** | **0** | **1** | **2** | **3** | **4** | **5** | **n** |
| Cdc45, 1.3 nM DDK,  –SDPGC  (Figure 2C – panel i)^a^ | 0.69  [0.66, 0.72] | 13.41  [13.36, 13.49] | 0.092  [0.090, 0.094] | 4.12  [4.03, 4.21] | 0.682  (± 0.001) | 0.222  (± 0.002) | 0.079  (± 0.001) | 0.0159  (± 0.0004) | - | - | 78 |
| Cdc45, 1.3 nM DDK,  +SDPGC  (Figure 2C – panel ii)^a^ | 0.31  [0.29, 0.32] | 11.00  [10.95, 11.05] | 0.101  [0.010, 0.103] | 4.19  [4.16, 4.23] | 0.560  (± 0.001) | 0.294  (± 0.001) | 0.116  (± 0.001) | 0.0262  (± 0.0004) | - | - | 131 |
| Cdc45, 1.3 nM DDK,  +SDPGC  (Figure 3C – panel i)^c^ | 0.33  [0.30,0.38] | 6.13  [6.00, 6.28] | 0.123  [0.116, 0.129] | 2.78  [2.69, 2.86] | 0.512  (± 0.004) | 0.295  (± 0.009) | 0.144  (± 0.004) | 0.040  (± 0.003) | - | - | 48 |
| Cdc45, 6.5 nM DDK,  +SDPGC  (Figure 3C – panel ii)^c^ | 0.346  [0.340, 0.353] | 5.79  [5.74, 5.84] | 0.310  [0.306, 0.314] | 2.10  [2.07, 2.12] | 0.295  (± 0.001) | 0.137  (± 0.004) | 0.216  (± 0.004) | 0.194  (± 0.002) | 0.109  (± 0.002) | 0.039  (± 0.001) | 87 |
| Cdc45, 6.5 nM DDK,  +SDPGC, -Sld3/7  (Figure 3 – panel iii)^c^ | 7  [4, 10] | 4.54  [4.39, 4.67] | 0.02  [0.01, 0.03] | 0.73  [0.60, 0.88] | 0.982  (± 0.001) | 0.017  (± 0.001) | - | - | - | - | 32 |
| 1:20 Cdc45, 1.3 nM DDK,  +SDPGC  (Figure 4 – supp 1A)^c^ | -0.96  [-0.98, -0.57] | 6.20  [6.16, 6.25] | 0.0003  [0^e^, 0.0033] | 3.12  [3.03, 3.18] | 0.934  (± 0.001) | 0.059  (± 0.001) | - | - | - | - | 40 |
| 1:20 Cdc45, 6.5 nM DDK,  +SDPGC  (Figure 4 – supp 1B)^c^ | 0.6  [0.4, 0.8] | 6.37  [6.27, 6.49] | 0.048  [0.042, 0.055] | 2.05  [1.94, 2.18] | 0.797  (± 0.002) | 0.169  (± 0.003) | 0.030  (± 0.002) | - | - | - | 72 |
| Cdc45, 6.5 nM DDK,  +SDPGC, Mcm2-7^6AD/E + ASP/Q^  (Figure 6C – panel iii)^c^ | 0.27  [0.24, 0.30] | 5.8  [5.6, 5.9] | 0.19  [0.18, 0.20] | 3.3  [3.2, 3.4] | 0.356  (± 0.005) | 0.272  (± 0.002) | 0.226  (± 0.002) | 0.107  (± 0.003) | 0.032  (± 0.001) | - | 43 |
| Cdc45, 6.5 nM DDK,  +SDPGC, Mcm2-7^4AD/E + ASP/Q^  (Figure 6C – panel iv)^c^ | 0.12  [0.08, 0.17] | 4.8  [4.6, 4.9] | 0.16  [0.15, 0.17] | 2.2  [2.1, 2.3] | 0.331  (± 0.004) | 0.34  (± 0.01) | 0.223  (± 0.003) | 0.083  (± 0.005) | 0.020  (± 0.002) | - | 33 |
| Cdc45, 6.5 nM DDK,  +SDPGC, Mcm2-7^Δ6N^  (Figure 6 – supp 1B)^c^ | 0.8  [0.3, 0.9] | 7.85  [7.56, 7.89] | 0.017  [0.012, 0.019] | 4.628  [4.485, 4.632] | 0.925  (± 0.003) | 0.064  (± 0.004) | 0.010  (± 0.003) | - | - | - | 89 |

^a^Continuous image acquisition protocol; see Materials and Methods.

^b^See Materials and Methods

^c^Alternating image acquisition protocol; see Materials and Methods.

^d^Values less than 0.01 not shown.

^e^Confidence interval lower limit uncertain because of minimal binding in this experiment (see Materials and Methods).

**Supplementary File 1b. Parameters used for fitting of GINS fluorescence-intensity histograms.**

| **Fit parameters^a^**  **[90% CI]** | | | | **Fraction of time that DNAs with loaded Mcm2-7^4SNAP549^ were bound by this number of GINS^SORT649^ molecules (± SE)^c^** | | | | | | |  |  |
| --- | --- | --- | --- | --- | --- | --- | --- | --- | --- | --- | --- | --- |
| **Condition**  **(Figure)** | ***q*** | ***s***  **(× 10^-3^)** | **λ** | ***x***  **(× 10^-3^)** | **0** | **1** | **2** | **3** | **4** | **5** | **6** | **n** |
| GINS, 1.3 nM DDK,  +SDPGC, On Mcm2-7  (Figure 5C – panel i)^b^ | 0.68  [0.56, 0.70] | 6.11  [6.06, 6.15] | 0.270  [0.231, 0.273] | 2.33  [2.29, 3.01] | 0.45  (± 0.01) | 0.14  (± 0.02) | 0.183  (± 0.006) | 0.136  (± 0.006) | 0.063  (± 0.006) | 0.019  (± 0.003) | - | 84 |
| GINS, 6.5 nM DDK,  +SDPGC, On Mcm2-7  (Figure 5C – panel ii)^b^ | 0.071  [0.070, 0.073] | 5.15  [5.05, 5.24] | 0.41  [0.40, 0.42] | 1.65  [1.62, 1.67] | 0.080  (± 0.002) | 0.078  (± 0.004) | 0.188  (± 0.005) | 0.258  (± 0.002) | 0.222  (± 0.003) | 0.123  (± 0.004) | 0.042  (± 0.002) | 57 |
| GINS, 6.5 nM DDK,  +SDPGC, On Mcm2-7, –Sld3/7  (Figure 5C – panel iii)^b^ | 1.35  [1.31, 1.39] | 4.94  [4.88, 5.01] | 0.155  [0.152, 0.159] | 2.25  [2.20, 2.30] | 0.684  (± 0.003) | 0.162  (± 0.002) | 0.105  (± 0.001) | 0.038  (± 0.001) | - | - | - | 49 |
| GINS, 6.5 nM DDK,  +SDPGC, On Mcm2-7, –Cdc45  (Figure 5C – panel iv)^b^ | 1.60  [1.59, 1.61 | 5.67  [5.62, 5.71] | 0.264  [0.262, 0.265] | 3.31  [3.30, 3.32] | 0.648  (± 0.001) | 0.095  (± 0.002) | 0.086  (± 0.001) | 0.039  (± 0.001) | 0.0110  (± 0.0003) | - | - | 79 |
| GINS, 6.5 nM DDK,  +SDPGC, Non-Mcm2-7  (Figure 5 – supp 1)^b^ | 1.55  [1.53, 1.58] | 5.35  [5.27, 5.42] | 0.351  [0.346, 0.357] | 2.13  [2.10, 2.16] | 0.621  (± 0.002) | 0.053  (± 0.001) | 0.101  (± 0.001) | 0.109  (± 0.001) | 0.074  (± 0.001) | 0.032  (± 0.001) | - | 84 |
| GINS, 6.5 nM DDK,  +SDPGC, On Mcm2-7, –Dpb11  (Figure 5 – supp 2)^b^ | 1  [-1, 3] | 3.4  [3.2, 3.5] | 0.0048  [0^d^, 0.0101] | 0.82  [0^d^, 0.96] | 0.983  (± 0.001) | 0.017  (± 0.001) | - | - | - | - | - | 32 |

^a^See Materials and Methods

^b^Alternating image acquisition protocol; see Materials and Methods.

^c^Values less than 0.01 not shown.

^d^Confidence interval lower limit uncertain because of minimal binding in this experiment (see Materials and Methods).
